# Supplementary figures and images for: Small extracellular vesicles derived from umbilical cord mesenchymal stem cells repair blood-spinal cord barrier disruption after spinal cord injury through down-regulation of Endothelin-1 in rats
Source: PeerJ. 2023 Oct 31;11:e16311. doi: 10.7717/peerj.16311 (PMC10624166; doi:10.7717/peerj.16311)

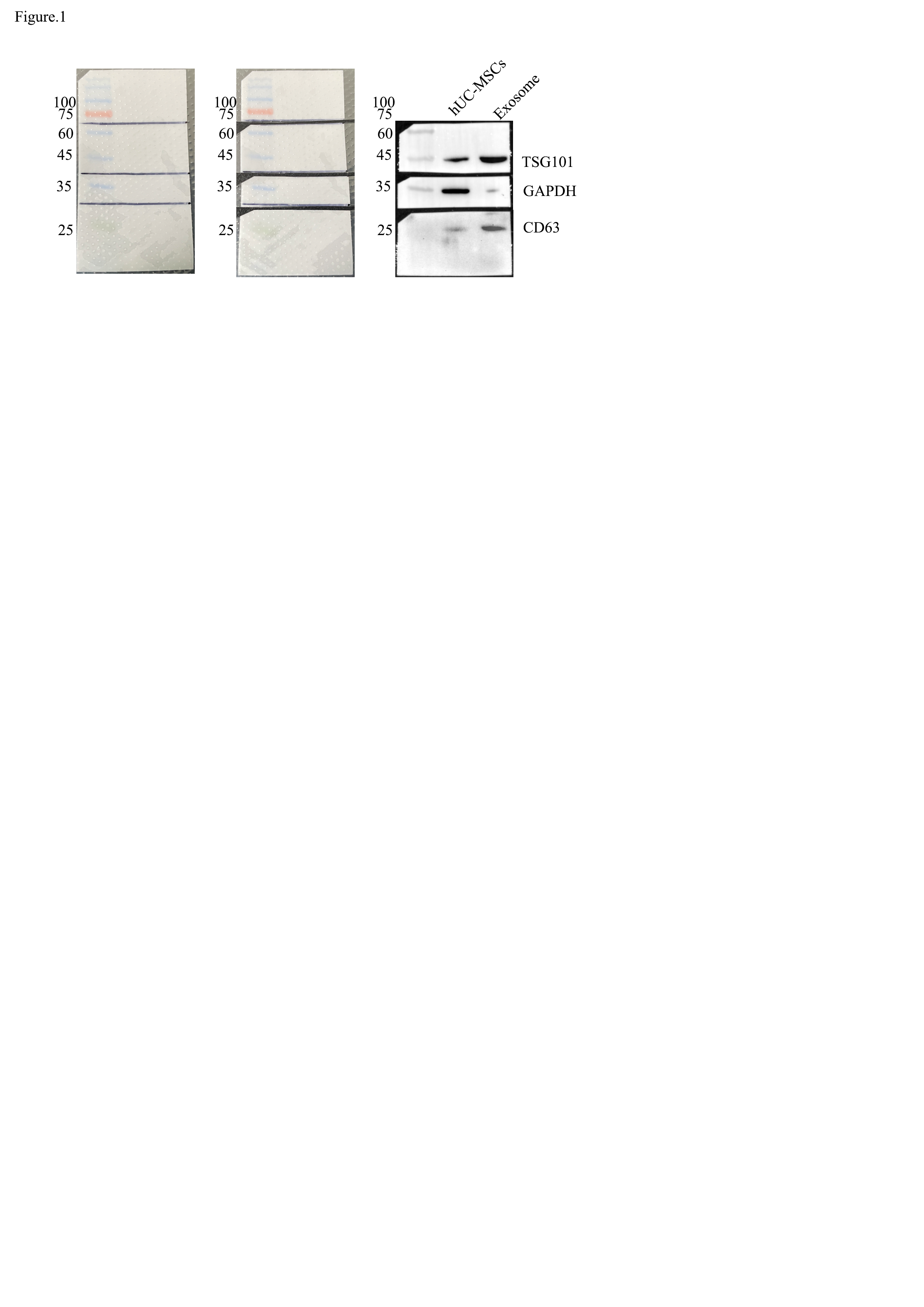

Supplement: Supplemental Information 3 [file peerj-11-16311-s003.zip › The raw data of Western blot/original gel_fig1.png]

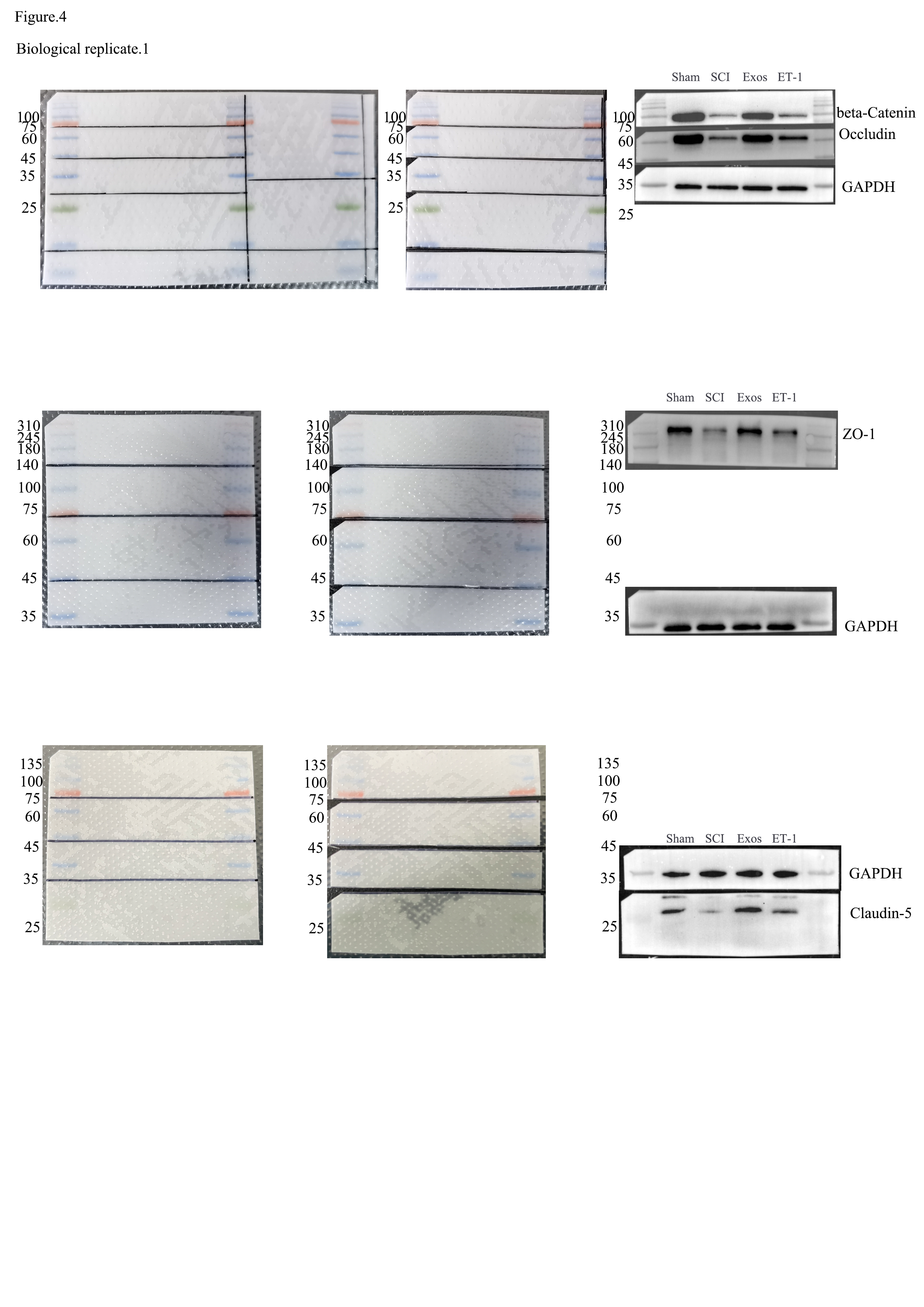

Supplement: Supplemental Information 3 [file peerj-11-16311-s003.zip › The raw data of Western blot/original gel_fig4_replicate1.png]

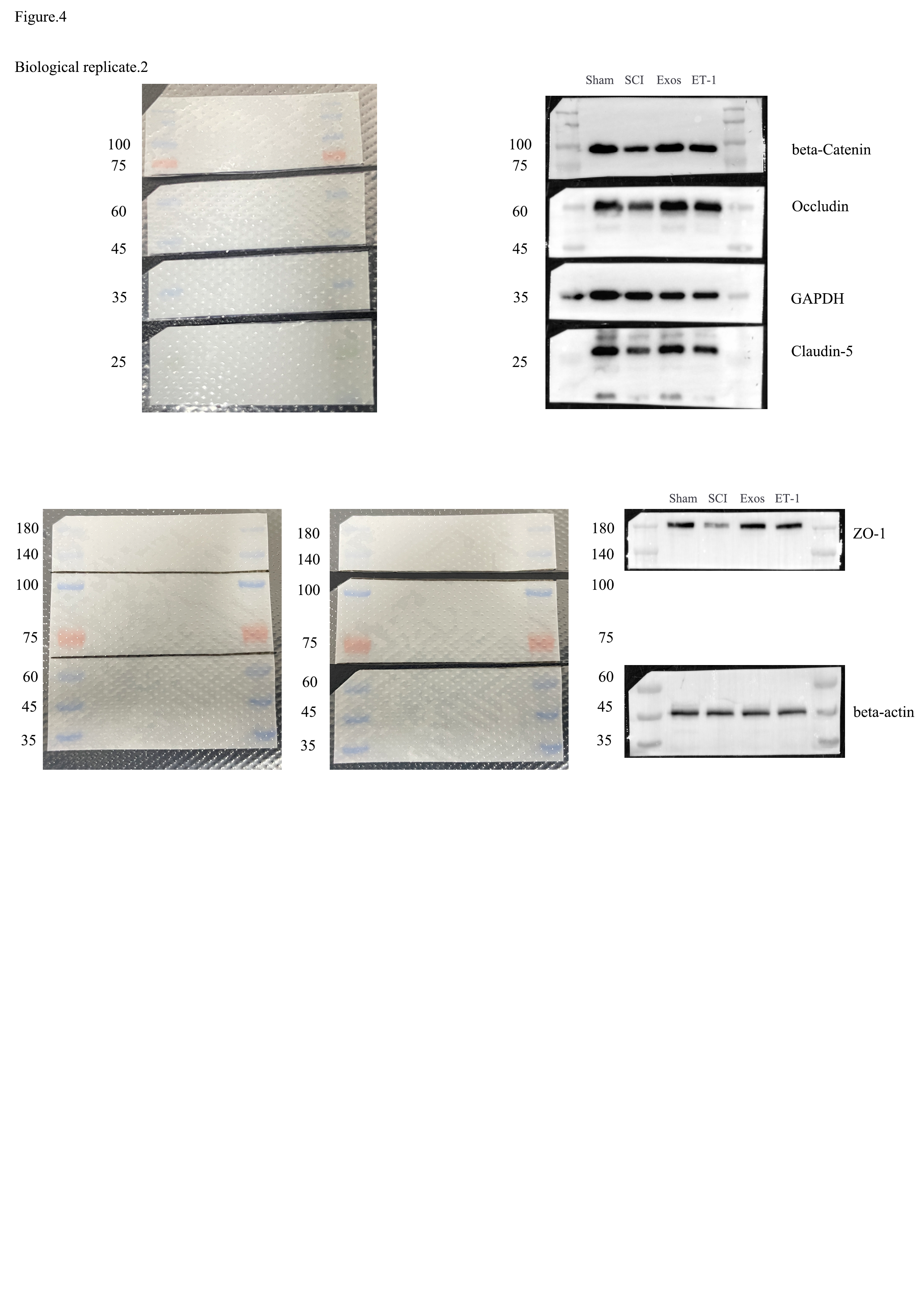

Supplement: Supplemental Information 3 [file peerj-11-16311-s003.zip › The raw data of Western blot/original gel_fig4_replicate2.png]

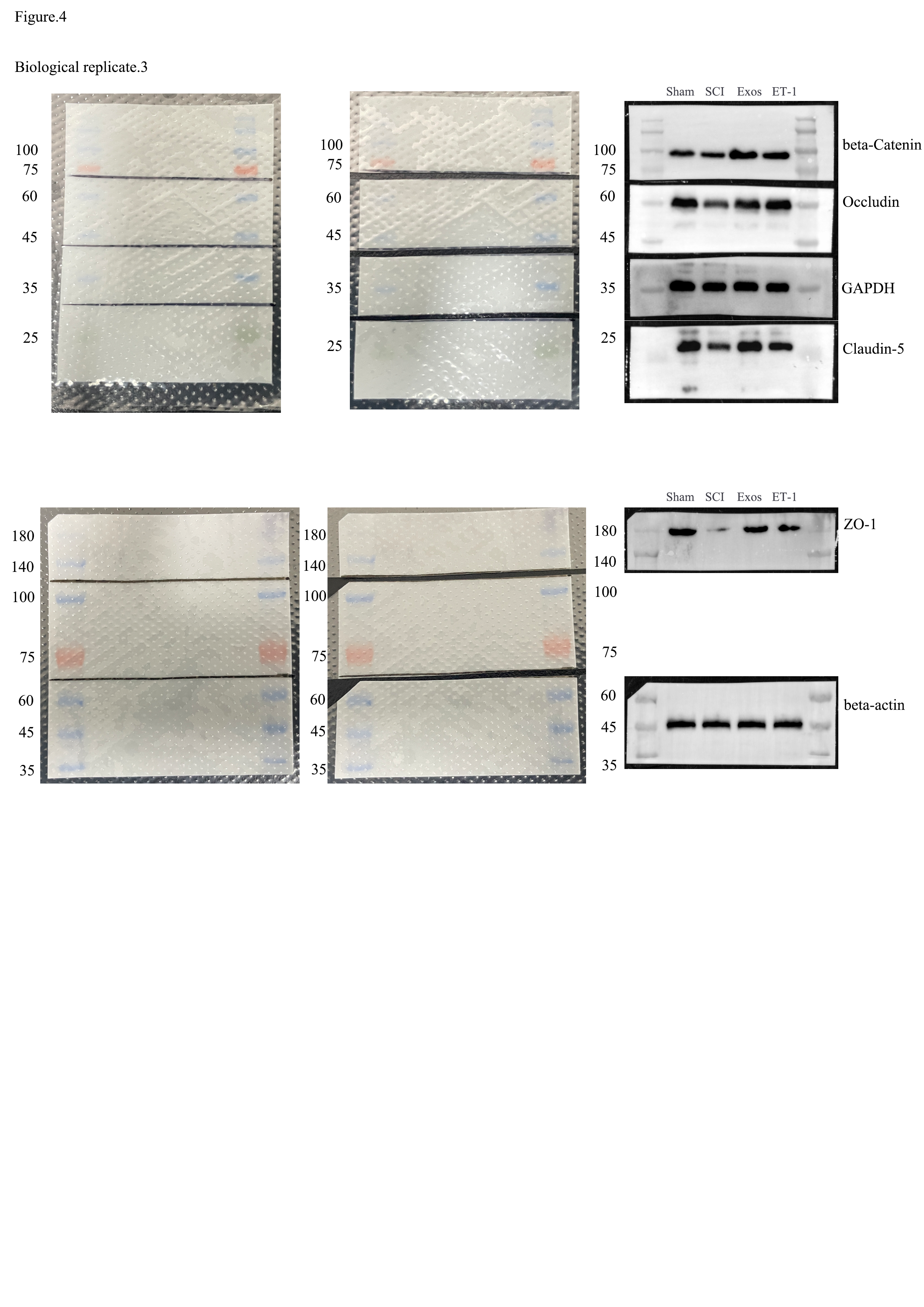

Supplement: Supplemental Information 3 [file peerj-11-16311-s003.zip › The raw data of Western blot/original gel_fig4_replicate3.png]

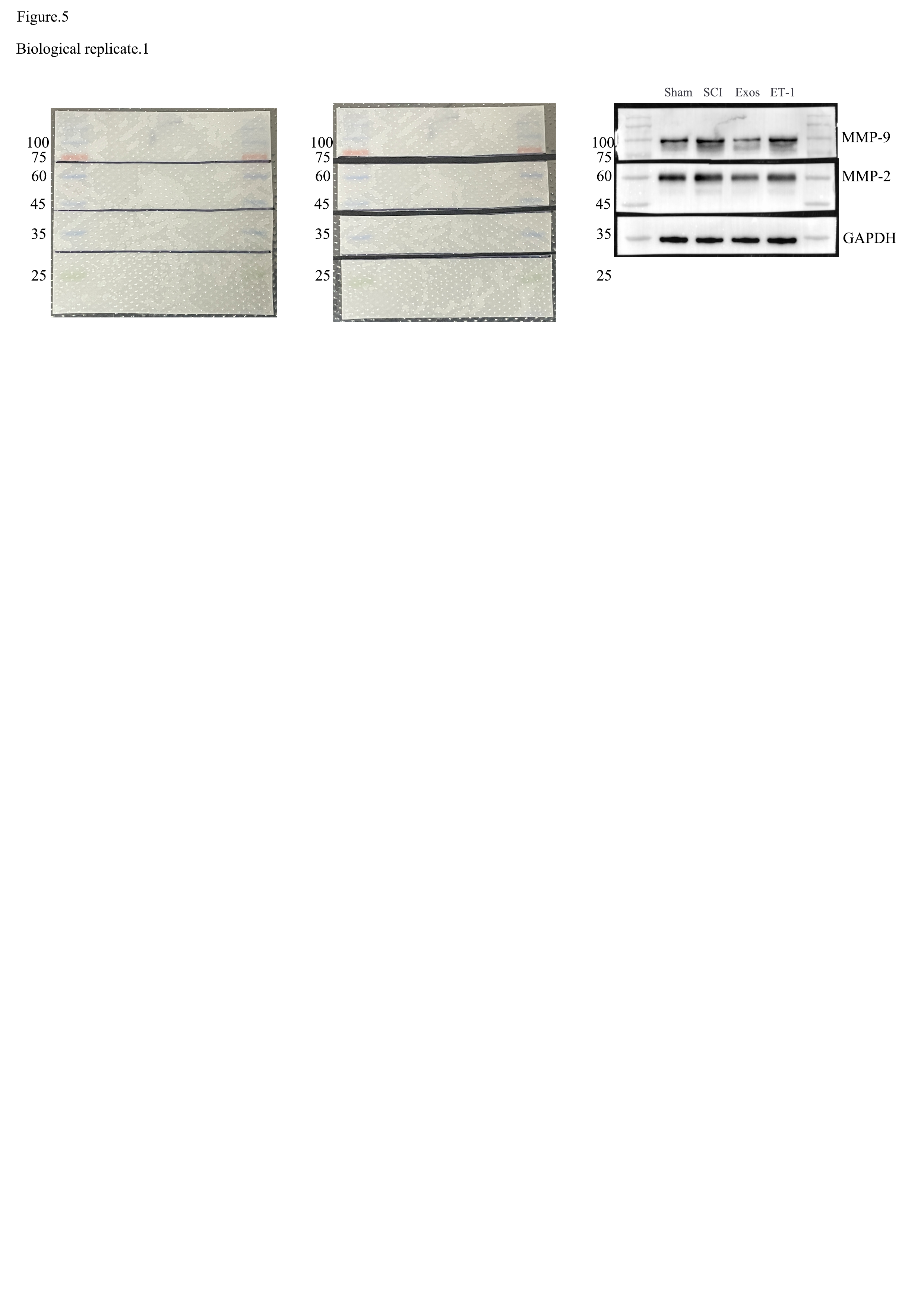

Supplement: Supplemental Information 3 [file peerj-11-16311-s003.zip › The raw data of Western blot/original gel_fig5_replicate1.png]

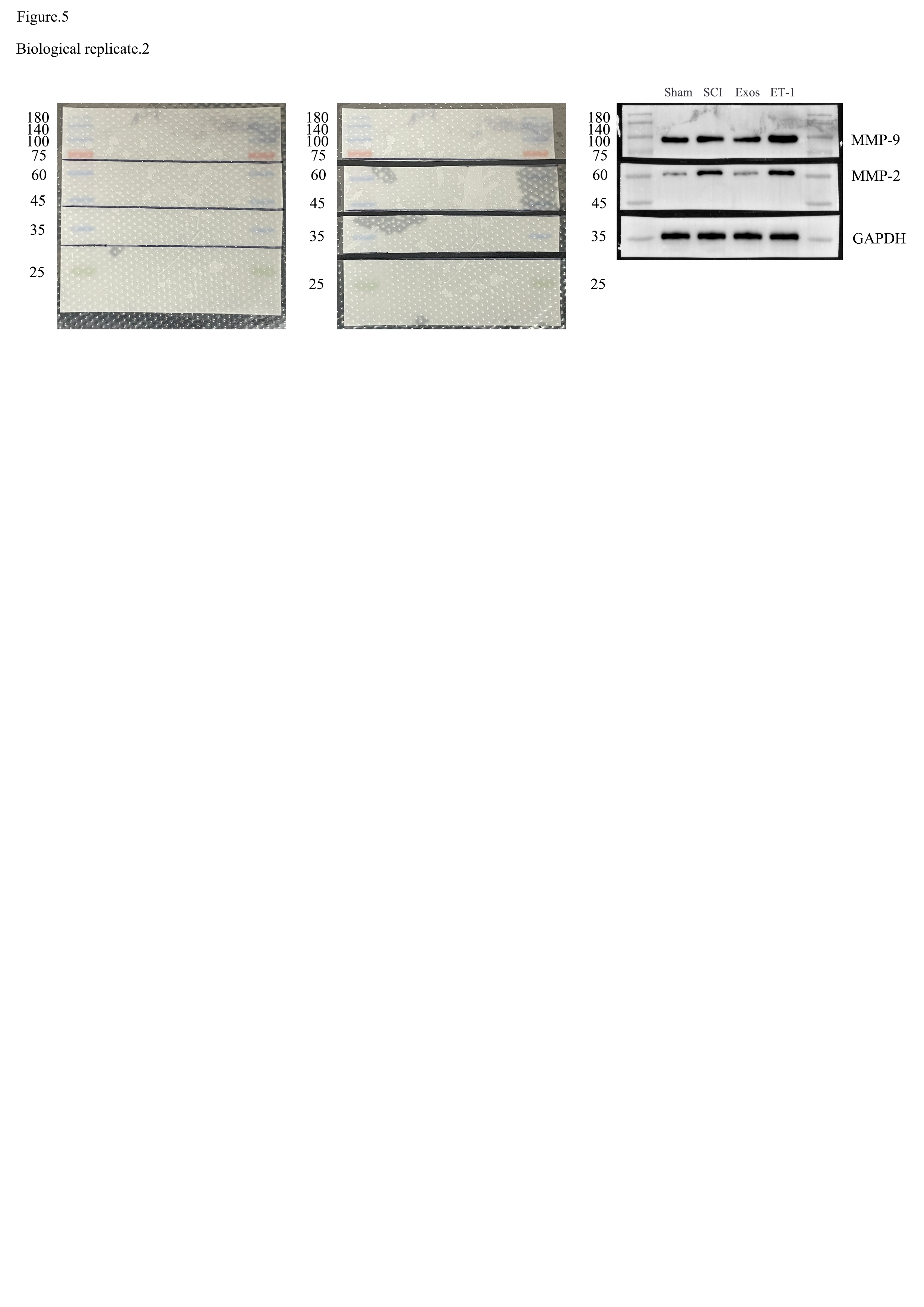

Supplement: Supplemental Information 3 [file peerj-11-16311-s003.zip › The raw data of Western blot/original gel_fig5_replicate2.png]

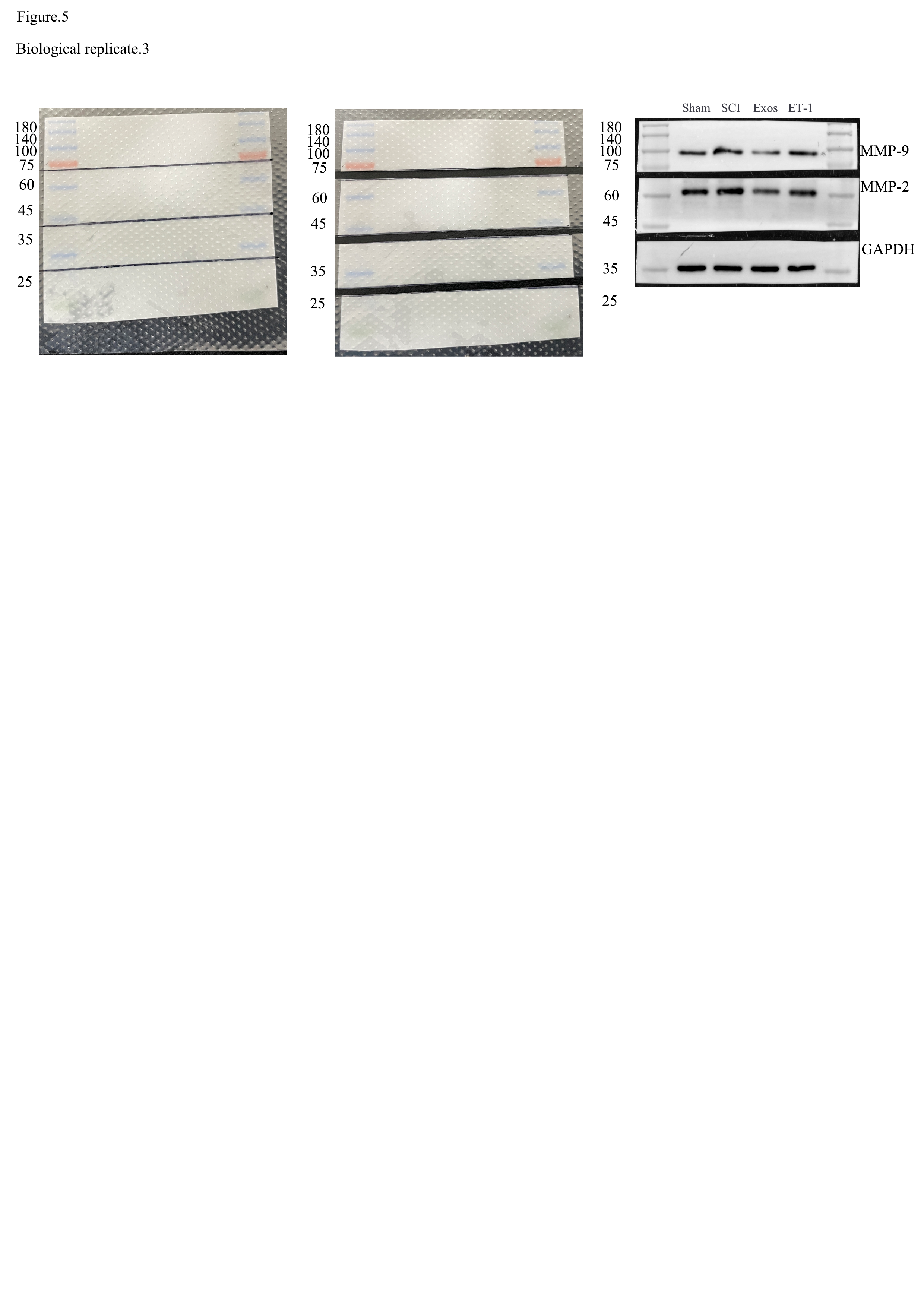

Supplement: Supplemental Information 3 [file peerj-11-16311-s003.zip › The raw data of Western blot/original gel_fig5_replicate3.png]

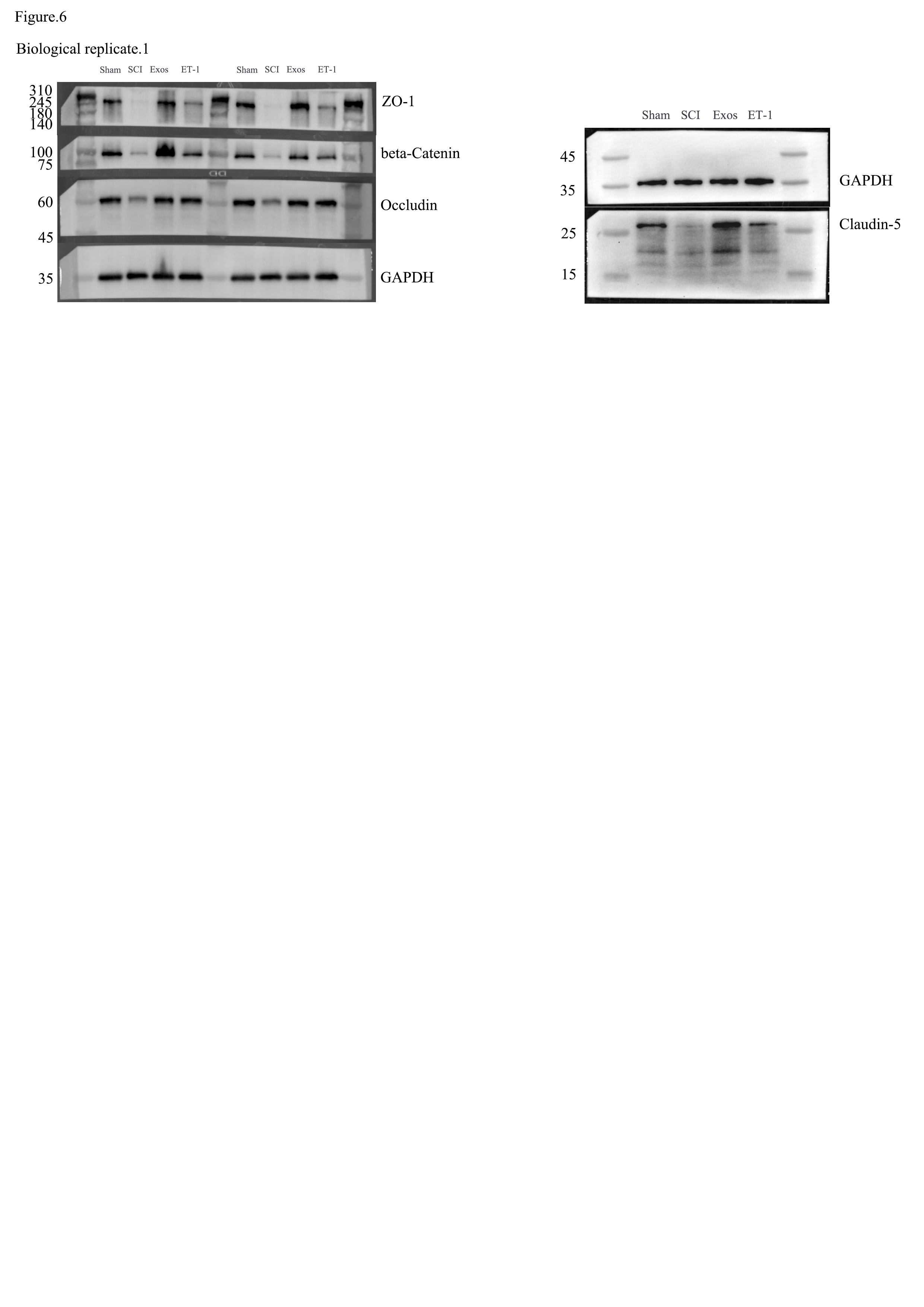

Supplement: Supplemental Information 3 [file peerj-11-16311-s003.zip › The raw data of Western blot/original gel_fig6_replicate1.png]

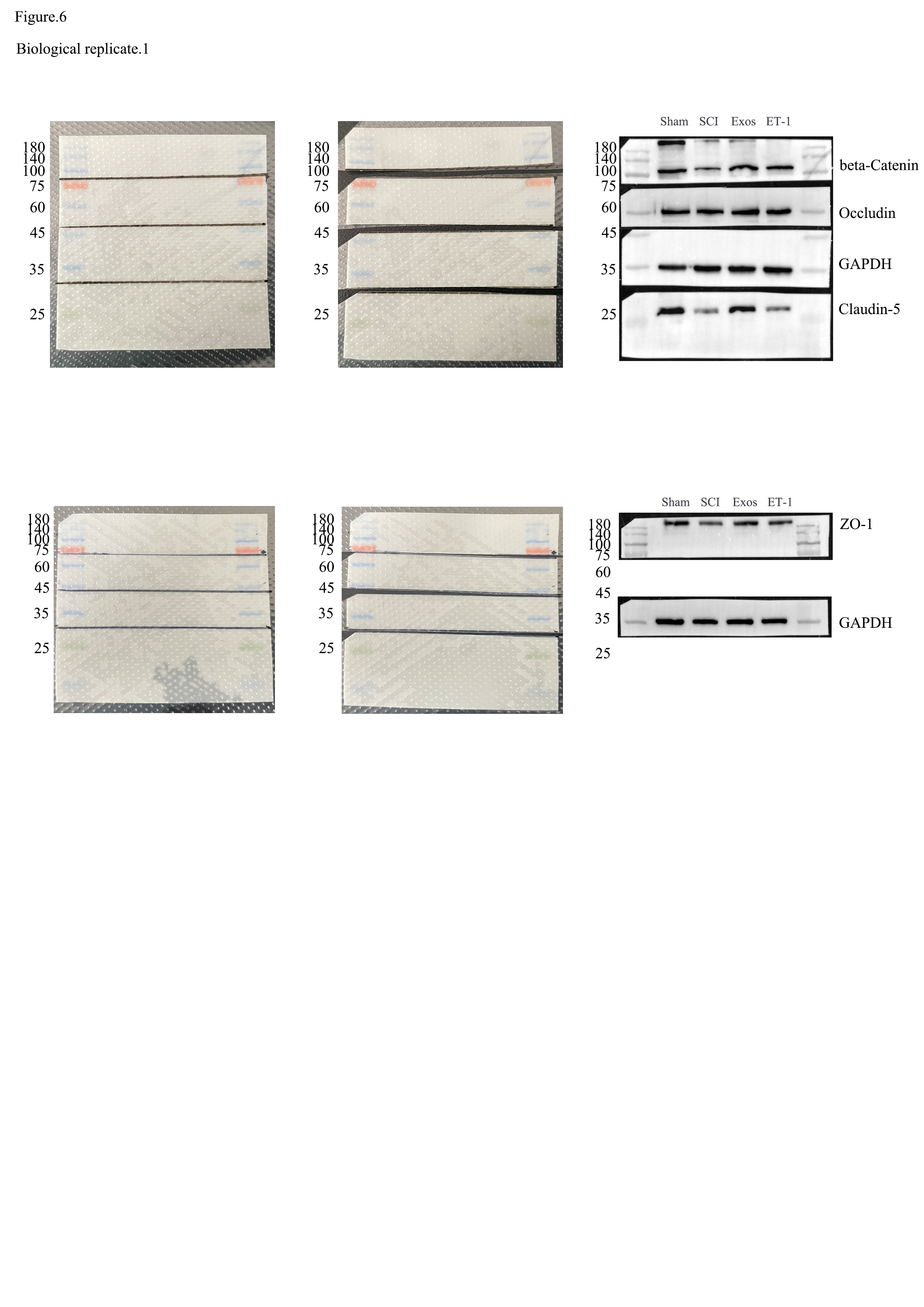

Supplement: Supplemental Information 3 [file peerj-11-16311-s003.zip › The raw data of Western blot/original gel_fig6_replicate2.png]

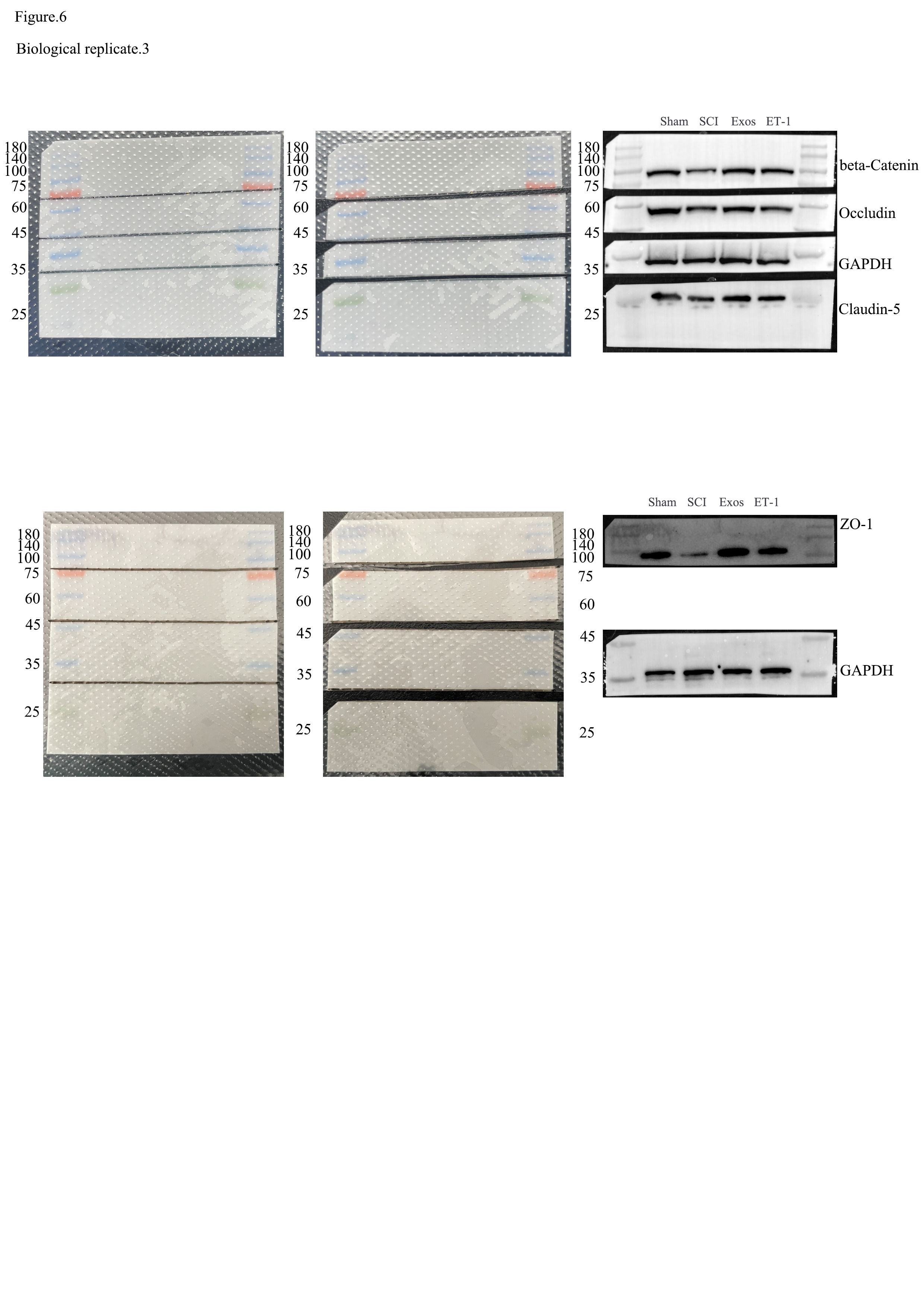

Supplement: Supplemental Information 3 [file peerj-11-16311-s003.zip › The raw data of Western blot/original gel_fig6_replicate3.png]
